# Effect of dalteparin, a low-molecular-weight heparin, as adjunctive therapy in patients with Kawasaki disease: a retrospective study

**DOI:** 10.1186/1471-2431-14-27

**Published:** 2014-01-30

**Authors:** Yasuji Inamo, Katsuya Saito, Maki Hasegawa, Rika Hayashi, Takahiro Nakamura, Osamu Abe, Teruaki Ishikawa, Yayoi Yoshino, Koji Hashimoto, Tatsuo Fuchigami

**Affiliations:** 1 Department of Pediatrics and Child Health, Nihon University School of Medicine, Tokyo, Japan

**Keywords:** Kawasaki disease, Low-molecular-weight heparin, Dalteparin, Coronary artery disease, Angiogenesis, IVIG

## Abstract

**Background:**

Dalteparin, a low-molecular-weight heparin, has anticoagulant and anti-angiogenic activity. This study investigated whether dalteparin reduced coronary artery lesion (CAL) prevalence, and resistance to intravenous immunoglobulin (IVIG) therapy in Kawasaki disease (KD).

**Methods:**

This retrospective study comprised two parts. In the first cohort, 126 patients with KD (68 male, 58 female; median age: 22 months, range: 1–67 months) admitted to Nihon University Nerima-Hikarigaoka Hospital from January 2004 to June 2008, received either dalteparin 75 IU/kg/day, IVIG 400 mg/kg/day for 5 consecutive days, and aspirin 30 mg/kg/day, or dalteparin 75 IU/kg/day and aspirin 30 mg/kg/day, until clinical improvement. Control data came from the 2005–6 Nationwide KD survey. In the second cohort, 112 patients with KD (59 male, 53 female; median age: 19 months, range: 1–66 months) admitted from June 2010 to February 2012, received either dalteparin 75 IU/kg/day, IVIG 2.0 g/kg over 12 h, and aspirin 30 mg/kg/day, or dalteparin 75 IU/kg/day and aspirin 30 mg/kg/day. Control data came from the 2009–10 Nationwide KD survey. No patients enrolled in the nationwide surveys received dalteparin. All patients at our institution were given dalteparin in their combination therapy.

**Results:**

A comparison of the first cohort with controls in the nationwide survey showed that the prevalence of initial administration of IVIG was 80.2% *versus* 86.0%; the rate of additional IVIG administration was 7.1% *versus* 14.0% (p = 0.03); CAL prevalence in the acute period was 4.8% *versus* 11.9% (p < 0.01); and the prevalence of cardiovascular sequelae was 0% *versus* 3.8% (p < 0.05). A comparison of the second cohort with controls in the nationwide survey showed that the rate of initial administration of IVIG was 92.9% *versus* 89.5%; the rate of additional IVIG administration was 8.9% *versus* 17.1% (p = 0.02); the prevalence of resistance to IVIG was 3.6% *versus* 14.9% (p < 0.001); and CAL prevalence in the acute period was 2.7% *versus* 8.6% (p = 0.03).

**Conclusions:**

This study found that adjunctive dalteparin was associated with a lower prevalence of IVIG resistance and CAL in young children with KD.

**Trial registration:**

UMIN-CTR: UMIN000010349.

## Background

High-dose intravenous immunoglobulin (IVIG), together with aspirin, is effective in resolving inflammation associated with Kawasaki disease (KD). IVIG also effectively reduces the occurrence of coronary artery lesions (CAL), but these still occur in 12% of patients treated with low-dose IVIG (400 mg/kg/day for 5 days) [1] and in 2.5% of patients treated with high-dose IVIG (2 g/kg/day) [2].

The Japan Kawasaki Disease Research Committee conducted the 21st Nationwide Survey of patients with KD who visited target hospitals for the treatment of acute KD during the 2-year period from January 2009 through December 2010 [3]. The medical institutions included in the survey were hospitals that specialized in pediatrics, and hospitals that had a total of 100 or more beds and a pediatric department.

Despite the effectiveness of IVIG treatment in reducing cardiovascular complications in KD, between 5% and 10% of treated patients develop CAL [3,4], which has led to the search for more effective drugs for the treatment of KD. Hence, we aimed to develop a more effective and safe regimen for the treatment of KD, regardless of the need for any additional rescue treatment that might include corticosteroids or anti-cytokine drugs.

Vascular endothelial growth factor (VEGF) plays an important role in maintaining vascular homeostasis. Dysregulation of VEGF activity as well as that of other angiopoietins contributes to the disruption of vascular homeostasis in KD [5]. High VEGF levels have been described in KD and have been implicated in the pathological findings observed in vascular tissue in KD [6-9]. Dalteparin is a low-molecular-weight form of heparin that inhibits both coagulation and angiogenesis, and is safe and easy to use in clinical practice. Treatment with dalteparin has been proposed to act by preserving endothelial function through inhibition of the production and activity of VEGF [10-12]. In Japan, dalteparin (75 units/kg/day continuous intravenous infusion) is recommended for the treatment of disseminated intravascular coagulation because it is associated with a lower bleeding tendency than unfractionated heparin (p < 0.05, Level 2b evidence) [13]. However, the same indication for dalteparin is not recommended in other countries. Indeed, dalteparin is only approved for intravenous use in hemodialysis and hemofiltration procedures in Canada, and not in the U.S.

Subcutaneous injection of dalteparin or enoxaparin is also available for treating venous thromboembolism (VTE), which is associated with heterozygous protein S or C deficiency and is a major secondary complication in advanced tertiary care of infants and children with critical illness. Thus, optimal prophylactic and treatment strategies for VTE are extremely important in children to avoid such complications. Subcutaneous enoxaparin is also indicated for severe coronary artery disease requiring long-term systemic anticoagulation after the acute phase of KD [14]. However, low-molecular-weight heparin, including enoxaparin and fondaparinux, is only available as a subcutaneous injection, thus we chose dalteparin for continuous intravenous infusion.

In the present study, patients with acute KD in our institution had initial treatment with a combination of dalteparin, aspirin, and/or IVIG. The aim of this retrospective and pilot study was to investigate whether dalteparin in the initial treatment phase is associated with a lower prevalence of CAL in patients with acute KD.

## Methods

### Ethics statement

**Figure 1:**
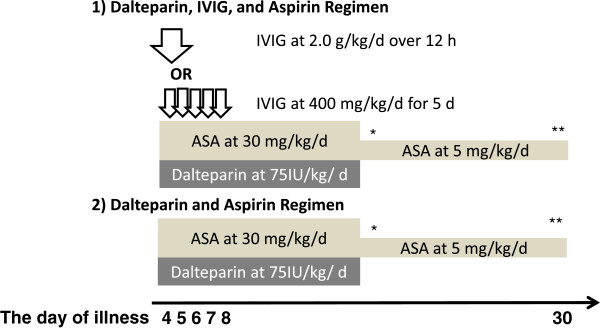
**The two regimens of dalteparin combination therapy.** *Aspirin (ASA) was decreased to 5 mg/kg/day if the patient was afebrile, clinically improving, and/or showed a CRP < 1.0 mg/dL. **Aspirin withdrawn after 1 month of illness unless there were cardiac sequelae. Dalteparin was administered until the patient was afebrile, clinically improving, and/or showed a CRP < 1.0 mg/dL. ***IVIG started on day 4 or 5 of illness (the first illness day was defined as the day of onset of fever >38°C).

The study protocol conformed to the Declaration of Helsinki and was approved by the Institute Review Board of Nihon University Nerima-Hikarigaoka Hospital (NUNH Hospital). All patients’ parents or guardians gave informed consent for treatment with dalteparin, aspirin, and IVIG (Figure 1). All clinical investigations were conducted according to the principles expressed in the Declaration of Helsinki.

### Diagnosis

The diagnostic criteria were compliant with the Diagnostic Guidelines for Kawasaki Disease (5th revision) [15].

### Data collection

All KD patients admitted to the NUNH Hospital, at which all authors formerly worked, were assigned initial treatment according to the Harada score, which is frequently used to predict increased risk of CAL before IVIG treatment in Japan. The Harada score comprises the following criteria: (1) white blood cell count > 12,000/mm^3^, (2) platelet count < 350,000/mm^3^, (3) C-reactive protein (CRP) concentration > 4.5 mg/dL, (4) hematocrit < 35%, (5) serum albumin concentration < 3.5 mg/dL, (6) age < 12 months, and (7) male sex. For KD patients with at least four of these seven attributes, aspirin and IVIG therapy is typically administered, and for patients with less than three attributes, only aspirin is administered [16].

There are currently no guidelines in any country that recommend withholding IVIG treatment in acute KD, thus we conventionally adopted the Harada score protocol whereby ASA alone is given to selected patients with KD in Japan. ASA alone is generally not recommended in acute KD, although the 19th and 21st Nationwide Surveys reported that ASA alone was administered to 14.0% and 10.0%, respectively, of patients with KD in Japan (Tables 1 and 2). It is also noteworthy that the group assigned to dalteparin and ASA alone in this study received non-standard therapy that is not currently recommended in any country, including Japan.

**Table 1:** Number and percentage of cases according to components of therapy in patients with KD at the NUNH Hospital in patients in the 19th Nationwide survey (2005–6) and the first cohort (low-dose IVIG)

	**19**^**th **^**Nationwide survey (2005–2006)**		**First cohort (2004–2008)**		**p-value**
Total	20,475	100.0	126	100.0	
Dosage of IVIG	Cases	%	Cases	%	
2 g/kg	11,612	56.7	3	2.1	0.001
1 g/kg/day × 2 d	4,800	23.4	3	2.1	0.001
400 mg/kg/day × 5 d	231	1.1	95	75.4	0.001
Various dosages of IVIG	879	4.3	0	0	0.01
Aspirin alone (no IVIG)	2,866	14.0	25	19.8	0.07
Dalteparin	0	0	126	100.0	
Age (months)	-*		22 (0–167)		
Male sex	11,892 (58.1%)		68 (54.0%)		0.37

KD: Kawasaki disease; NUNH Hospital: Nihon University Nerima-Hikarigaoka Hospital; IVIG: intravenous immunoglobulin.

Dalteparin was administered until the patient was afebrile, clinically improving, and/or showed a C-reactive protein (CRP) < 1.0 mg/dL.

Data are median (range), n (%).

*The highest prevalence was observed among those aged 6–8 months [17].

**Table 2:** Number and percentage of cases according to components of therapy in patients with KD at the NUNH Hospital between the 21st Nationwide survey (2009–10) and the second cohort (high-dose IVIG)

	**21**^**st **^**Nationwide survey (2009–10)**		**Second cohort (2010–12)**		**p-value**
Total	23,730	100.0	112	100.0	
Dosage of IVIG	Cases	%	Cases	%	
2 g/kg	17,547	74.0	85	75.9	0.75
1 g/kg/day × 2 d	2,803	11.8	5	4.5	0.01
400 mg/kg/day × 5 d	20	0.08	4	3.6	0.001
Various dosages of IVIG	868	3.65	0	0	0.04
Aspirin alone (no IVIG)	2,492	10.5	18	16.1	0.06
Dalteparin	0	0	112	100.0	
Age (months)	-*		19 (0–66)		
Male sex	13,515 (57.0%)		59 (52.7%)		0.39

Dalteparin was administered until the patient was afebrile, clinically improving, and/or showed a CRP < 1.0 mg/dL.

Data are median (range), n (%).

*The prevalence was highest among children aged 6–11 months [3].

The IVIG dose was chosen by the treating clinician, with variations across both cohorts from 400 mg/kg/day for 5 days, to 2 g/kg over 12 h for 1 day, or 1 g/kg over 12 h for 2 days.

The study design was retrospective and comprised two parts. Subjects in the first cohort (n = 126 patients with KD) were admitted to the NUNH Hospital between January 2004 and June 2008. The control data were sourced from the 19th Nationwide Survey of KD performed from January 2005 through December 2006 [17]. The patients were treated with either dalteparin at 75 IU/kg/day as a continuous intravenous infusion until clinical improvement along with low-dose IVIG (400 mg/kg/day for 5 consecutive days) and aspirin at 30 mg/kg/day or a combination of dalteparin and aspirin at the same doses. IVIG and dalteparin infusions were given simultaneously, with the patients requiring a second intravenous access line for IVIG. The dose of aspirin was decreased to 5 mg/kg/day if the patient was afebrile, clinically improving, and/or had CRP < 1.0 mg/dL. Dalteparin was administered until the patient was afebrile, clinically improving, and/or had CRP < 1.0 mg/dL (Table 1).

Subjects in the second cohort (n = 112 patients with KD) were admitted to NUNH Hospital between June 2010 and February 2012. Control data were sourced from the 21st Nationwide Survey of KD performed from January 2009 through December 2010. The patients were treated with either dalteparin at 75 IU/kg/day as a continuous intravenous infusion and high-dose IVIG (2 or 1 g/kg over 12 h for 1 or 2 days, respectively) and aspirin 30 mg/kg/day or a combination of dalteparin and aspirin at the same doses. IVIG and dalteparin infusions were given simultaneously, with the patients requiring a second intravenous access line for IVIG. The aspirin dose was decreased to 5 mg/kg/day in patients who were afebrile, clinically improving, and/or had a CRP < 1.0 mg/dL. Dalteparin was administered until the patient was afebrile, clinically improving, and/or had CRP < 1.0 mg/dL (Table 2).

A survey of CAL was performed using echocardiography in all patients at follow-up, and within 3 months of discharge. None of the patients were administered corticosteroids. Although there was no information about aspirin administration in the nationwide surveys, we considered that aspirin was an anchor drug and it was administered to all controls with KD. There was no report of dalteparin administration in the nationwide surveys and therefore it was assumed that dalteparin was not administered to controls with KD.

### Definition of coronary artery lesions

The coronary arteries of patients were regularly assessed by pediatricians using two-dimensional echography. Acute cardiac lesions were defined as those that developed within 1 month of onset (acute lesions); cardiac sequelae were defined as those that persisted beyond 1 month after onset [17].

We reviewed these records within 3 months from the onset of KD. CAL was diagnosed in patients in accordance with the following Japanese Ministry of Health criteria: an internal lumen diameter > 3.0 mm in children < 5 years of age or > 4.0 mm in children ≥ 5 years of age, an internal segment diameter at least 1.5 times larger than that of an adjacent segment, or an irregular lumen [18]. Although some KD patients present with CAL at diagnosis [19], we did not find any cases before initial treatment in either cohort.

### Requirements for additional IVIG therapy

Additional IVIG therapy was provided when patients had persistent fever > 38°C (axillary temperature) lasting 24 h after the completion of the initial treatment [20,21].

### Resistance to IVIG therapy

Resistance of KD to IVIG was defined as the persistence or recurrence of fever > 38°C (axillary temperature) at least 48 h after the end of the additional IVIG therapy [22].

### Statistical analysis

We calculated the required sample size based on the assumption that IVIG plus dalteparin would lower the prevalence of acute-phase CAL from 12.0% to 4.0% (first cohort) and from 9.0% to 2.0% (second cohort). With a two-sided test, an α value of 0.05, a power of 80%, and a total sample of 101 (first cohort) and 94 patients (second cohort) would be needed.

Data are presented as a percentage for categorical variables. Baseline characteristics and components of treatment were compared between the dalteparin group and the control group using the chi-square test for categorical variables. For all analyses, a two-sided p < 0.05 was considered indicative of statistical significance. The odds ratio was calculated as crude odds. The adjusted odds ratio for dosage of initial IVIG therapy in both groups was obtained using the Mantel-Haenstzel method, while the adjusted odds ratio for acute-phase CAL, cardiac sequelae, and additional IVIG therapy was not analyzed because the nationwide surveys contained no dosage information for the various initial IVIG treatments.

### Coagulation parameters

Monitoring of prothrombin time and activated partial thromboplastin time was not necessary. Monitoring of dalteparin therapy is only possible using an anti-factor Xa assay. Anti-Xa levels might be useful to monitor effects in patients with severe renal dysfunction, abnormal coagulation parameters, or bleeding. We did not monitor anti-factor Xa levels in our cohort studies.

## Results

### Patient population

In the first cohort, there were 126 KD patients (68 male, 58 female; median age: 22 months, range: 1–167 months) (Table 1). A total of 20,475 patients with KD (11,892 male, 8,583 female) were reported in the 19th Nationwide Survey, including 17,613 patients with initial IVIG treatment, 2,860 patients with additional IVIG treatment, 2,446 patients with CAL identified during the acute phase of the disease, and 712 patients with cardiac sequelae after 1 month from onset of KD. No subjects who were enrolled in the nationwide surveys received dalteparin. There were no data on patient characteristics, demographics, and disease severity in the control population [17], which is a limitation of this study.

In the second cohort, there were 112 KD patients (59 were male, 53 were female; median age: 19 months, range: 1–66 months) (Table 2). A total of 23,730 patients with KD (13,515 were male, 10,215 were female) were reported in the 21st Nationwide Survey, including 21,247 patients with initial IVIG treatment, 4,049 patients with additional IVIG treatment, 3,532 patients with resistance to IVIG, 2,034 patients with CAL who were identified during the acute phase of disease, and 695 patients with cardiac sequelae after 1 month from onset of KD. No subjects who were enrolled in the nationwide surveys received dalteparin. Again, there were no data on patient characteristics, demographics, and disease severity in the survey [3].

### Comparison between the first cohort and the 19th nationwide survey

Initial IVIG therapy was used in 80.2% of patients in the first cohort *versus* 86.0% in the survey (p = 0.06) (Table 3). Additional IVIG therapy was used in 7.1% of patients in the first cohort *versus* 14.0% in the survey (p = 0.027). The occurrence of CAL in the first cohort was 4.8% *versus* 11.9% in the survey (p = 0.013), and cardiac sequelae occurred in 0% *versus* 3.8% (p = 0.047) of patients. The rate of resistance to IVIG therapy in the first cohort was 7.9% but no information on IVIG resistance was reported in the 19th Nationwide Survey (Table 3). There were no adverse events associated with dalteparin in the first cohort.

**Table 3:** Comparison between the 19th Nationwide survey and the first cohort

	**19th Nationwide survey n; %**	**First cohort group n; %**	**p-value**	**Odds ratio**	**95% CI**
Total	20,475; 100.0	126; 100		(crude)	
Initial IVIG therapy	17,613; 86.0	101; 80.2	0.06	0.91	0.53–1.57
			0.38*	1.07*	0.88 – 1.30*
Acute phase CAL	2,446; 11.9	6; 4.8	< 0.01	2.71	1.19– 6.17
Cardiac sequelae	772; 3.8	0; 0	< 0.05	0	^‡^NA
Additional IVIG therapy	2,860; 14.0	9; 7.1	0.03	2.08	1.06–4.13

CI: confidence interval; CAL: coronary artery lesions.

^‡^NA, unable to be calculated because the number contained “0.”

*Adjusted odds ratio for dosage of initial IVIG therapy in both groups was obtained using the Mantel-Haenstzel method adjusted for various initial dosages of intravenous immunoglobulin (2 g/kg, 1 g/kg, 400 mg/kg, or various dosages of IVIG). Adjusted odds ratio for acute-phase CAL, cardiac sequelae, and additional IVIG therapy were not analyzed because there were no descriptions of dosages of various initial IVIG in the nationwide surveys.

### Comparison between the second cohort and 21st nationwide survey

As shown in Table 4, initial IVIG therapy was administered to 92.9% of patients in the second cohort *versus* 89.5% in the survey (p = 0.24). Additional IVIG therapy was given to 8.9% of patients in the second cohort *versus* 17.1% in the survey (p = 0.022). Similarly, the occurrence of CAL in the second cohort was 2.7% *versus* 8.6% in the survey (p = 0.025), and the occurrence of cardiac sequelae was 0.89% *versus* 2.9% (p = 0.32). The rate of resistance to IVIG therapy was 3.6% in the second cohort *versus* 14.9% in the survey (p < 0.001) (Table 4). There were no adverse events associated with dalteparin in the second cohort.

**Table 4:** Comparison between the 21st Nationwide survey and the second cohort

	**21st Nationwide survey n; %**	**Second cohort group n; %**	**p-value**	**Odds ratio**	**95% CI**
Total	23,730; 100.0	112; 100.0		(crude)	
Initial IVIG therapy	21,247; 89.5	104; 92.9	0.24	0.46	0.23–0.95
			0.31*	1.20*	0.86–1.69*
Acute phase CAL	2,044; 8.6	3; 2.7	0.03	3.43	1.09–10.79
Cardiac sequelae	696; 2.9	1; 0.9	0.32	3.35	0.47–24.06
Additional IVIG therapy	4,049; 17.1	10; 8.9	0.02	2.10	1.10–4.02
Resistance for IVIG therapy	3,532; 14.9	4; 3.6	< 0.001	5.16	1.09–14.00

*Adjusted odds ratio for dosage of initial IVIG therapy in both groups was obtained using the Mantel-Haenstzel method adjusted for various initial dosages of intravenous immunoglobulin (2 g/kg, 1 g/kg, 400 mg/kg, or various dosages of IVIG). Adjusted odds ratio for acute-phase CAL, cardiac sequelae, and additional IVIG therapy were not analyzed because there were no descriptions of dosages of various initial IVIG in the nationwide surveys.

The occurrence of CAL, as well as the rate of additional IVIG therapy and rate of resistance to IVIG therapy in the second cohort group were markedly lower than in the control group (Table 4).

### Comparison between the first cohort and the second cohort

There was a significant increase in the proportion of patients receiving initial IVIG therapy between the first and second cohort groups (p < 0.005); but no other comparisons were not significantly different.

## Discussion

Our cohorts showed reduced occurrence of acute-phase CAL and cardiac sequelae compared with patients in either nationwide survey. The occurrence of cardiac sequelae in patients on IVIG at 400 mg/kg/day for 5 days was approximately 12%, whereas the occurrence in patients given dalteparin in addition to IVIG at 400 mg/kg/day for 5 days was 0%. An additional study has reported that the occurrence of cardiac sequelae in patients on IVIG at 2 g/kg/day was 2.5% [2].

The rate of resistance to IVIG therapy in KD in the second cohort was markedly lower compared with that reported in the 21^st^ Nationwide Survey. Thus, the results from the second cohort support the conclusion that dalteparin promotes the effectiveness of IVIG therapy in acute KD. This suggests that dalteparin in combination with IVIG may be more efficacious than IVIG alone.

The antiangiogenic activity of dalteparin, together with its known anticoagulant activity provides the basis for the beneficial clinical effects observed in KD. Moreover, dalteparin therapy seems to be well tolerated and is not associated with significant side effects [13].

### Limitations

The present study at a single institution was limited by its nonrandomized, retrospective nature. It should be noted that such data might underestimate the true prevalence of CAL, cardiac sequelae, and IVIG unresponsiveness in patients with KD. Therefore, all potential sources of bias and confounders cannot be excluded from any study. A further potential limitation of this study was the use of the 19th and 21st Nationwide Surveys for epidemiological control data because these surveys were not truly matched populations with respect to our own cohorts. There were differences in the IVIG dosing regimen between the first cohort and controls (Table 1), but not between the second cohort and controls (Table 2). In addition, the analysis did not account for potential confounders, e.g., disease severity, age, sex, fever, and laboratory data.

Other limitations include the non-standardized echo readings without a core laboratory and single examiner, and lack of laboratory studies to monitor anti-factor Xa levels to support the safety of dalteparin in this setting. In addition, we left the choice of dose of IVIG to each doctor, and there may have been other unobserved or unmeasured confounders associated with individualized clinical decision-making.

## Conclusions

This study found that concomitant dalteparin therapy was associated with a lower prevalence of IVIG resistance and CAL in KD. A multicenter randomized trial should be performed to collect prospective data on KD patients, and to further determine the efficacy of adjunctive therapy with dalteparin in KD patients.

## Competing interests

The authors have no potential conflict of interest. We state that no honorarium, grant, or other form of payment was given to anyone to produce the manuscript.

## Authors’ contributions

YI conceived and designed the study, analyzed the data, and wrote the paper. All authors contributed equally to performance of the study. All authors read and approved the final manuscript.

## Pre-publication history

The pre-publication history for this paper can be accessed here:

http://www.biomedcentral.com/1471-2431/14/27/prepub
